# Inter-limb asymmetry across multiple soccer tasks varies with maturity status in young players: a cross-sectional study

**DOI:** 10.5114/biolsport.2026.154159

**Published:** 2025-09-16

**Authors:** Hamza Marzouki, Bilel Cherni, Alâa Edine Sassi, Anissa Bouassida, Ezdine Bouhlel, Yung-Sheng Chen, Karim Chamari

**Affiliations:** 1 Research Unit: Sport sciences, Health and Movement, University of Jendouba, Kef, Tunisia; 2 High Institute of Sport and Physical Education of Kef, University of Jendouba, Tunisia; 3 Research Laboratory, Exercise Physiology and Physiopathology: from Integrated to Molecular “Biology, Medicine and Health” (LR19ES09), Faculty of Medicine of Sousse, University of Sousse, Tunisia; 4 Exercise and Health Sciences, University of Taipei, Taiwan; 5 Tanyu Research Laboratory, Taipei, Taiwan; 6 Exercise and Health Promotion Association, New Taipei City, Taiwan; 7 High Institute of Sport and Physical Education of Ksar-Said, University of Manouba, Tunisia; 8 Naufar, Wellness and Recovery Center, Doha, Qatar

**Keywords:** Growth, Motor asymmetry, Change of direction, Balance, Dribbling deficit, Youth athletes

## Abstract

Biological maturation is one of the determinants influencing physical performance, yet its impact on asymmetry in soccer-specific tasks remains poorly understood. This cross-sectional study investigated the effects of biological maturation on inter-limb asymmetry in lower limb muscle volume (LLMV), linear and changeof-direction (COD) sprints (with and without the ball), jump performance, and dynamic balance. The agreement between asymmetry indices in dribbling tasks was assessed using Kappa coefficients to determine the consistency of asymmetry direction across tasks. Eighty-three young male soccer players (Pre-PHV: n = 42, Circa-PHV: n = 30, Post-PHV: n = 11) performed (1) 10-m sprint and 90° COD tests with- (S10_drib_ and COD_drib_, respectively) and without the ball (S10_run_ and COD_run_, respectively), (2) bilateral and unilateral countermovement jumps, and (3) the Y-balance test. Asymmetry indices were calculated for all performance measures. The Pre-PHV players showed greater asymmetry in S10_drib_, COD_drib_, and COD_drib_-Deficit than Circa- and Post-PHV (p < 0.05). The S10_drib_-Deficit asymmetry was higher in Pre-PHV players than that of Circa-PHV players (p = 0.038), while Post-PHV players had lower COD_drib_ asymmetry than Circa-PHV players (p = 0.016). Balance asymmetry was greater in Post-PHV players than their counterparts (p < 0.05). Kappa coefficients showed fair to moderate agreement between asymmetry directions in S10_drib_ and COD_drib_, and between their respective deficits, indicating task-specific asymmetry consistency. These findings underscore the need for maturation-specific training strategies, with early-maturing players benefiting from targeted neuromuscular training, while older players should focus on balance and sport-specific drills to manage asymmetry and injury prevention.

## INTRODUCTION

Physical performance assessment in young soccer players has gained attention, particularly regarding rapid changes of direction (COD) and ball control [1, 2]. Soccer requires physical and technical skills, including unilateral and bilateral jumps, sprints, and COD maneuvers, performed with and without the ball [3]. These tasks provide insights into players’ physiological and biomechanical characteristics, making them essential for training and monitoring [4].

Biological maturation introduces variability in young athletes’ physical capacities, influencing strength, speed, coordination, and lower limb muscle volume—key factors for optimal soccer performance [1, 5]. Lower limb muscle volume (LLMV), crucial for strength and power, plays a pivotal role in jumping, sprinting, and dynamic balance [6]. However, its impact on performance asymmetries remains unclear. Performance asymmetry, defined as the difference between limbs, affects both performance and injury risk [7, 8, 9] and is commonly assessed through unilateral jumps and COD sprints, which replicate match demands [6]. Asymmetries exceeding 10–15% have been associated with compromised movement efficiency and a higher risk of non-contact injuries (e.g. anterior cruciate ligament (ACL) ruptures or hamstring strains. This highlights the importance of monitoring these imbalances for injury prevention in applied settings, particularly during high-intensity unilateral actions such as sprinting, jumping, and cutting [7, 10]. Despite growing research, the relationship between biological maturation and asymmetries— particularly in linear and COD sprints, with and without the ball— remains underexplored.

The ability to change direction quickly, especially while dribbling, is crucial for evading opponents and creating scoring opportunities [3, 11, 12]. Dribbling and COD deficits provide a functional measure of directional asymmetry beyond sprint times [13]. Yet how biological maturation influences these deficits remains unclear, highlighting a gap in understanding asymmetry development and consistency. Another critical aspect of soccer performance is dynamic balance, which refers to maintaining postural control during movement [13]. This skill integrates strength, coordination, and neuromuscular control, vital in asymmetrical actions such as cutting and dribbling [13]. Studies have emphasised that asymmetries in balance tasks can fluctuate during late adolescence, particularly when rapid physical growth temporarily affects neuromuscular control [14]. Likewise, it has been reported that elite young soccer players exhibit smaller inter-limb asymmetries and superior body composition compared to their non-elite counterparts, emphasising the importance of addressing asymmetry in athletic development [5].

Research on inter-limb asymmetries has examined their directionality and consistency [14]. Investigating whether asymmetry in one task, such as COD, aligns with asymmetry in another, such as dribbling, has important training implications [3]. Quantifying this agreement using statistical measures (e.g., kappa coefficients) allows practitioners to determine the reliability and relevance of these assessments for training [5]. If asymmetries shift with maturation, training programs can be adjusted accordingly. Recognising consistent asymmetries across tasks could refine training priorities.

To our knowledge, no study has simultaneously investigated the influence of biological maturation on asymmetries across multiple soccer-specific performance (e.g., linear and COD sprints with/without the ball, dynamic balance, and LLMV). Also, information regarding the agreement between asymmetry indices across soccer-specific tasks is less explored. This comprehensive approach offers novel insight into the task specificity and developmental trajectory of asymmetry in youth soccer. We therefore investigated the impact of biological maturation on performance asymmetries in young soccer players. We examined whether maturation status (Pre-, Circa-, or Post-PHV) affects asymmetry in LLMV, linear and COD sprints (with and without the ball), jumping, and dynamic balance. Additionally, we assessed the agreement between asymmetries in linear dribbling, COD dribbling, and their deficits across maturation stages. We hypothesised that inter-limb asymmetries in soccer-specific tasks would be greater in Pre-PHV players compared to Circa- and Post-PHV players, and that the agreement between asymmetry directions across tasks would vary due to maturation status.

## MATERIALS AND METHODS

### Participants

Prior to participant recruitment, a sample size estimation was conducted using G∗Power software (version 3.1.9.4, University of Kiel, Kiel, Germany) [15]. The study design [(analysis of variance (ANOVA) test with fixed effects, omnibus, one-way] was used as the basis for the estimation. Effect sizes for the calculation were derived from tabulated data in prior research [16]. The results indicated that 66 participants were required (f = 0.40 and actual power = 81.8%) to detect differences, assuming a Type I error of 0.05 and a Type II error of 0.20 (statistical power = 80%). This calculation focused on the primary ANOVA comparison adjusted for multiple comparisons, maintaining a family-wise error rate of p ≤ 0.05. Thus, 83 male youth soccer players from the same regional-level soccer clubs volunteered to participate in this study to avoid the dropout risk. The participants were categorised into three age groups: under 13 (n = 42), under 15 (n = 30) and under 17 (n = 11). Biological maturity status was assessed using the method proposed by Mirwald et al. [17]. The maturity offset was calculated using the following formula: Maturity offset = -9.236 + (0.0002708 × leg length × sitting height) + (-0.001663 × age × leg length) + (0.007216 × age × sitting height) + (0.02292 × body weight/height ratio). This calculation enabled the classification of participants into three groups based on PHV offset: Pre-PHV (-3 to > -1 years), Circa-PHV (-1 to +1 years), and Post-PHV (> +1 to +3 years). Inclusion criteria were: (1) a minimum of two years of soccer training experience; and (2) no severe musculoskeletal injuries (defined as those requiring surgical intervention or resulting in > 4 weeks of training/match absence) in the lower extremities (knee, ankle, or hip joints) within the previous 12 months, and (3) no mild to moderate injuries within a month (defined as those causing < 4 weeks of training absence) [18]. All participants were screened and were safe from injuries prior to preliminary testing. They had been involved in competitive soccer for at least 3–4 years in the Pre-PHV group, ~5 years in the Circa-PHV group, and 6–8 years in the Post-PHV group, respectively. Participants’ characteristics of biological maturity status are presented in Table 1. All participants competed regularly in regional championships. Their weekly training microcycle included four sessions (~70 to 90 min each) and one official match on Sundays. Parental informed consent was obtained prior to their inclusion in the study. The research was approved by the Institutional Ethics Committee (approval number: 016/2023; date of approval: October 09, 2023) and conducted by the Declaration of Helsinki.

**TABLE 1:** Participants’ anthropometric and maturity-related characteristics.

	Pre-PHV (n = 42)	Circa-PHV (n = 30)	Post-PHV (n = 11)
Age (yrs)	12.7 ± 0.3	14.6 ± 0.5	16.6 ± 0.3
PHV (yrs)	-2.3 ± 0.5	-0.4 ± 0.4	1.2 ± 0.1
APHV (yrs)	15.0 ± 0.5	14.9 ± 0.7	15.4 ± 0.4
Height (cm)	163.3 ± 10.3	178.2 ± 5.3	185.1 ± 2.7
Sitting height (cm)	76.2 ± 4.5	85.5 ± 4.2	89.6 ± 1.4
Total leg length (cm)	82.1 ± 5.6	92.7 ± 3.8	95.5 ± 2.0
Body mass (kg)	47.4 ± 10.8	62.4 ± 11.3	72.2 ± 11.8
BMI (kg · m^2^)	17.6 ± 2.6	19.6 ± 2.8	21.1 ± 3.3
BF (%)	15.2 ± 2.9	16.2 ± 5.4	15.7 ± 4.6
LLMV Right (l)	5.02 ± 1.08	6.66 ± 1.99	7.01 ± 1.81
LLMV Left (l)	5.03 ± 1.09	6.46 ± 1.52	6.87 ± 1.70
AI% LLMV (%)	9.5 ± 9.7	8.2 ± 8.0	10.7 ± 6.7

All values are expressed in mean ± SD. PHV: peak height velocity; APHV: age at peak height velocity; BMI: body mass index; BF: body fat mass; LLMV: lower limb muscle volume; AI%: asymmetry index percentage.

### Experimental Design

The current study adopted a cross-sectional design (between-subject comparative design and agreement) to examine whether biological maturity status affects asymmetry during linear and COD sprinting with and without the ball, vertical jumping, and dynamic balance. The experiment was conducted within the competitive season (i.e., from early January to late February 2024) and lasted 3 weeks for each group. During the first week, all participants attended two separate familiarisation sessions (~48 h apart). The second week was devoted to assessing anthropometric parameters. During the third week, participants conducted the following measurements: linear 10-m sprints with (S10_drib_) and without (S10_run_) the ball, a 10-m sprint (i.e., 5 + 5 m) with a 90º COD with (COD_drib_) and without (COD_run_) the ball, bilateral and unilateral countermovement jumps (CMJ), and the Y-balance test (see protocol below). The same raters performed all measurements.

All physical tests were conducted (1) ~48 h after the most recent weekly match to minimise the influence of fatigue and (2) under similar standardised conditions (synthetic grass soccer field; ambient temperature: 9º to 16º; humidity: 68 to 74%) with consistent verbal encouragements. Testing occurred over two separate days (on Tuesday and Thursday, from 18:00 to 20:00 h). On the first physical testing day, participants were assessed for (1) S10_drib_, (2) S10_run_, (3) bilateral, and (4) unilateral CMJ performances. On the second day, they performed (1) COD_drib_, (2) COD_run_, (3) and dynamic balance performances. Each physical testing session began with a ~12-min warm-up exercises that included jogging, dynamic stretching, and intense exercises (e.g., short sprints, skipping and bilateral and unilateral jumping). All tests involved two valid maximal trials with each foot (right, then left) in alternating order, separated by a ~2-min passive rest interval. The best record of each test was recorded for data analysis. A 3-min passive recovery was given between tests. Participants were instructed to maintain their normal intake of food and fluids the day before the tests. They were also asked to refrain from intense physical activity ~24 h before testing and to avoid consuming ergogenic drinks and food at least 2 h before testing, respectively [19].

### Procedures

#### Anthropometric Parameters Assessment

Body mass (kg) was measured with an accuracy of 0.1 kg using a digital scale (OHAUS, Florhman Park, NJ, USA). Height, sittingheight, and total leg length (LL) were recorded with a stadiometer to the nearest 0.1 cm (Seca model 213, Germany). Body mass index was calculated as body mass (kg) × height (m^2^). The biceps, triceps, subscapular, and suprailiac skinfolds were measured at traditional sites to estimate body density [20, 21] and percent body fat [22].

Five circumferences were measured on each leg (right and left) at 90º to the longitudinal axis: at the top of the thigh, mid-thigh, immediately below the patella, the maximum calf, and the minimum immediately above the ankle. These measurements were taken using a standardised anthropometric kit (Harpenden, Sweden). Leg length (*L*) was measured in cm from the midpoint of a line joining the uppermost circumference to the iliac crest, down to the minimum circumference above the ankle. The femoral intercondylar diameter of each leg was also recorded using a sliding caliper. Additionally, four skinfolds readings were taken on the quadriceps (front and back of mid-thigh) and calf (back and outside) of each leg, using a calibrated standard caliper (Harpenden/Holtain Calipers, Crosswell, Crymych, Pembrokeshire, UK). All measurements were performed by the same investigator following the techniques recommended by Weiner and Lourie [23].

The total volume of the lower limb was calculated using the formula: (∑*C*^2^) × *L*/62.8, where *C*^2^ is the square of an individual circumference measurement, while *L* is the corresponding lower limb length [24]. Fat volume was calculated as (∑*C*/5) × (∑*S*/2*n*) × *L*, where ∑*C* is the sum of the five circumferences, ∑*S* is the sum of skinfolds measured over the lower limb, and *n* is the number of skinfold readings for the lower limb. Bone volume was determined from the femoral intercondylar diameter, corrected for overlying fat. A preliminary study of radiographs suggested that in the leg, the average bone radius was 23.5% of the corrected femoral intercondylar diameter. Bone volume was then calculated using the formula: 3.14 × *R*^2^ × *L*, where *R* is the average bone radius for the lower limb. Finally, Muscle volume of the lower limb in liter (MVLL) was calculated as total lower limb volume – (fat volume + bone volume) [25].

#### Linear Sprint with and without Ball Assessment

To assess linear sprint performances, participants performed the S10_run_ and S10_drib_ tests in an all-out mode. Sprint times were recorded using a series of paired photocells (Globus, Microgate, Bolzano, Italy). The photocells were placed at a height of 0.2 m at the starting position, with a marker for the front foot positioned 0.2 m behind this point, and at a height of 1 m at the 10-m line. In the S10_drib_ test, participants dribbled a ball over a 10-m distance, with a minimum of three touches to control a ball. They were required to use one side of their foot for ball control during the test. Any trial was repeated if the ball was lost, went out of control, or if fewer than three touches were applied.

#### Change of Direction with and without the Ball Assessment

**FIG. 1:**
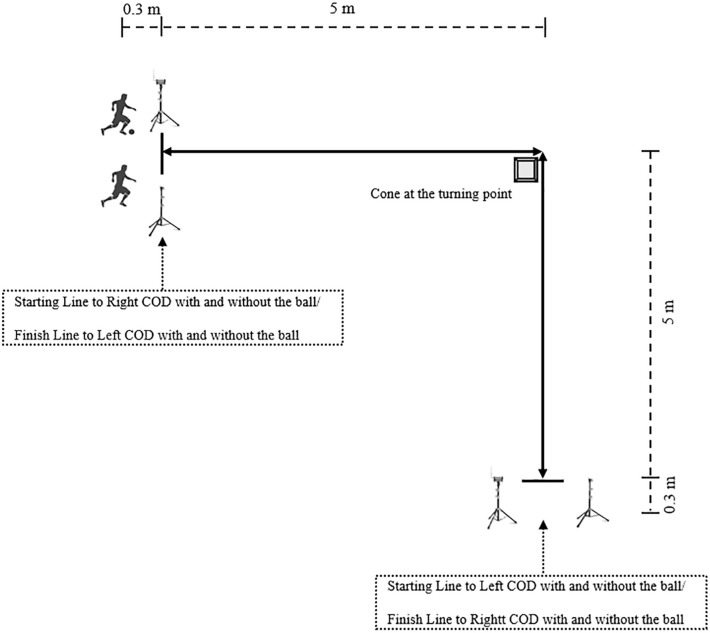
Diagram of the 90° change of direction (COD) test: black silhouettes represent COD with and without the ball, respectively.

A 10-m sprint (5 + 5 m) with a 90º COD, performed with and without the ball in an all-out mode, was conducted as described by Trecroci et al. [3]. All participants were instructed to perform the test in both directions (right and left) (Figure 1). For the performance evaluation (s), two paired photocells (Globus, Microgate, Bolzano, Italy) were placed—one at the start and the other at the end of the route—at a height of 0.75 m and with a 1.5-m distance between them, with a marker for the front foot positioned 0.3 m behind the starting line. For COD_drib_, participants were required to dribble the ball around the cone with a minimum of two touches (using the same foot) along each 5-m segment. The ball had to remain within close proximity throughout the maneuver. A trial was considered invalid and repeated if the cone was touched, the ball strayed beyond control, or the touch requirements were not met. For COD_run_, they were instructed to change direction around the cone using the same sidestep technique during each trial to avoid variability caused by differing COD execution techniques. If a participant hit or touched the cone at the turning point, the trial was stopped, and they were asked to repeat the test [26].

#### Bilateral and Unilateral Jumps Assessment

The bilateral CMJ without arm swing was used to assess jump height (cm), following the method described by Bosco et al. [27] and measured using an infrared jump system (Optojump; Microgate, Bolzano, Italy). Participants were required to start in a normal bipedal stance, squat with their legs bent at 90º, and immediately jump vertically as high as possible. To eliminate the influence of arm swing, participants were instructed to place their hands on their hips throughout the test. Knees and ankles were required to be fully extended on takeoff and during tiptoe landing.

The procedure for the unilateral CMJ was similar to that of the bilateral CMJ, except that participants were instructed to jump on a single leg while flexing the opposite leg to 90° at the hip and knee during the jump. This applied to both leg testing. A jump was considered invalid if improper technique was observed, such as failing to keep hands on the hips or failing to land on the same leg.

#### Dynamic Balance Assessment

The Y-balance test was adopted to evaluate the dynamic balance, following the method described by Plisky et al. [28]. The maximal reach distance (cm) for each leg and reach direction was recorded for further analysis. The composite score for each leg (%) was calculated and retained for analysis [28].

#### Dribbling and COD deficits

The dribble deficit was calculated by subtracting the total time for the dribbling trial from the total time for the corresponding nondribbling trial in both the linear sprint performance test and the COD test [29, 30]. The COD deficit was calculated by subtracting the S10_run_ total time from the COD_run_ total time [3].

#### Asymmetric Index Calculation

For all measured variables, the asymmetry index (AI%) was calculated using the following formula [31]: AI% = [100/(higher/faster side)] × (lower/slower side) × -1 +100.

### Statistical analysis

Descriptive statistics were reported as mean ± standard deviation (SD) or median ± interquartile range. The reliability of the physical tests (i.e., intraclass correlation coefficient [ICC], coefficient of variation [CV]) was calculated using the results from the familiarisation sessions and those obtained during the third week. The Shapiro-Wilk test was performed to test for normality. Since several key asymmetry variables violated the assumption of normality, and given the unequal sample sizes across the maturity groups, parametric tests were not appropriate. Therefore, the Kruskal-Wallis analysis of variance was applied to examine differences in asymmetry scores. Posthoc comparisons between groups were conducted using the Mann-Whitney U test to control for multiple comparisons while maintaining statistical robustness. Pearson’s r was employed to estimate the magnitude of significant findings as effect size (ES), with the thresholds defined as follows: 0.1–0.29 (small), 0.3–0.49 (medium), and 0.5 and above (large) [32]. Kappa coefficients were calculated to assess the level of agreement consistently favoring the same side (direction of asymmetry) between right and left dichotomous variables. These comparisons included the AI% values from linear and COD tasks (i.e., S10_drib_, COD_drib_, S10_drib_-Deficit, and COD_drib_-Deficit). This approach was selected because the Kappa coefficient measures the proportion of agreement between two methods while accounting for chance agreement [33]. Levels of agreement were interpreted according to Viera and Garrett [34]: < 0 (less than chance), > 0.01–0.20 (slight), > 0.20–0.40 (fair), > 0.40–0.60 (moderate), > 0.60–0.80 (substantial), > 0.80–0.99 (almost perfect), and 1 (perfect). These levels were used to determine whether asymmetry direction remained consistent across soccer-specific tasks. Statistical analyses were performed using SPSS version 26 for Windows (IBM Corp, Armonk, NY, USA), and significance was set at p ≤ 0.05.

## RESULTS

The descriptive statistics of each measured outcome are shown in Tables 1 and 2. The ICCs between the test-retest measurements ranged from 0.900 to 0.995 for all the physical measures, indicating excellent agreement between trials (Table 2).

**TABLE 2:** Descriptive statistics of physical performance measures.

	Pre-PHV (n = 42)	Circa-PHV (n = 30)	Post-PHV (n = 11)	ICC	CV (%)
CMJ (cm)	22.1 ± 4.6	28.5 ± 6.1	29.5 ± 9.5	0.975	5.90
CMJ Right (cm)	12.5 ± 2.4	14.6 ± 3.3	15.8 ± 4.6	0.962	5.84
CMJ Left (cm)	12.0 ± 2.1	13.7 ± 2.7	13.6 ± 2.6	0.930	7.03
AI% CMJ (%)	10.1 ± 6.1	10.9 ± 8.6	12.7 ± 8.9		
S10_run_ Right (s)	1.92 ± 0.12	1.86 ± 0.10	1.83 ± 0.08	0.966	1.59
S10_run_ Left (s)	1.93 ± 0.10	1.88 ± 0.09	1.84 ± 0.07	0.951	1.58
AI% S10_run_ (%)	-2.4 ± 1.1	-2.8 ± 1.2	-2.9 ± 1.2		
S10_drib_ Right (s)	2.46 ± 0.28	2.32 ± 0.21	2.13 ± 0.17	0.900	2.06
S10_drib_ Left (s)	2.53 ± 0.26	2.34 ± 0.20	2.15 ± 0.19	0.978	2.07
AI S10_drib_ (%)	-8.7 ± 5.7	-4.9 ± 1.7	-4.0 ± 1.5		
S10_drib_-Deficit Right (s)	0.54 ± 0.20	0.47 ± 0.14	0.30 ± 0.13		
S10_drib_-Deficit Left (s)	0.60 ± 0.21	0.46 ± 0.14	0.31 ± 0.14		
AI% S10_drib_-Deficit (%)	28.7 ± 20.0	15.8 ± 7.9	16.5 ± 8.9		
COD_run_ Right (s)	2.66 ± 0.31	2.46 ± 0.22	2.34 ± 0.20	0.983	1.96
COD_run_ Left (s)	2.74 ± 0.33	2.48 ± 0.25	2.39 ± 0.22	0.923	4.61
AI% COD_run_ (%)	-4.3 ± 2.4	-3.2 ± 1.9	-3.2 ± 1.4		
COD_run_-Deficit Right (s)	0.74 ± 0.29	0.60 ± 0.16	0.51 ± 0.19		
COD_run_-Deficit Left (s)	0.81 ± 0.31	0.60 ± 0.21	0.55 ± 0.20		
AI% COD_run_-Deficit (%)	14.3 ± 11.2	13.9 ± 9.1	10.0 ± 12.8		
COD_drib_ Right (s)	3.22 ± 0.32	2.99 ± 0.22	2.92 ± 0.28	0.917	4.21
COD_drib_ Left (s)	3.34 ± 0.38	3.03 ± 0.30	2.99 ± 0.21	0.985	1.89
AI% COD_drib_ (%)	-10.3 ± 4.1	-6.4 ± 2.2	-4.8 ± 2.0		
COD_drib_-Deficit Right (s)	0.56 ± 0.17	0.53 ± 0.11	0.58 ± 0.16		
COD_drib_-Deficit Left (s)	0.60 ± 0.26	0.55 ± 0.14	0.60 ± 0.15		
AI% COD_drib_-Deficit (%)	34.8 ± 16.0	19.1 ± 10.7	13.8 ± 8.9		
Balance Right (%)	81.2 ± 9.5	83.9 ± 7.7	81.3 ± 6.8	0.995	1.00
Balance Left (%)	81.0 ± 9.7	83.8 ± 7.1	79.5 ± 5.5	0.994	0.98
AI% Balance (%)	2.6 ± 2.4	2.4 ± 2.0	4.3 ± 2.7		

All values are expressed in mean ± SD. Negative AI% values represent better performance on the right side in sprint and COD tasks. PHV: peak height velocity; AI%: asymmetry index percentage; CMJ: bilateral countermovement jump; S10run: linear sprint of 10 m without the ball; S10drib: linear sprint of 10 m with the ball; CODrun: change of direction without the ball; CODdrib: change of direction with the ball; ICC: intraclass correlation coefficient; CV: coefficient of variation.

Table 3 shows the differences between biological maturity statuses. Pre-PHV players showed higher asymmetry in S10_drib_ (Circa-PHV: p = 0.013, ES = 0.291; Post-PHV: p = 0.004, ES = 0.395), COD_drib_ (Circa-PHV: p < 0.0001, ES = 0.535; Post-PHV: p < 0.0001, ES = 0.520) and COD_drib_-Deficit (Circa-PHV: p < 0.0001, ES = 0.522; Post-PHV: p < 0.0001, ES = 0.518) than their counterparts. Additionally, the S10_drib_-Deficit showed a significantly higher asymmetry in Pre-PHV players compared to Circa-PHV players (p = 0.038; ES = 0.324). The Post-PHV players showed significantly lower asymmetry in COD_drib_ in comparison to Circa-PHV players (p = 0.016; ES = 0.248). A significantly higher asymmetry in balance was reported for the Post-PHV players compared to those of Pre- (p = 0.012; ES = 0.344) and Circa-PHV groups (p = 0.019; ES = 0.368).

**TABLE 3:** Between-biological maturity-group differences.

	Pre-PHV (n = 42)	Circa-PHV (n = 30)	Post-PHV (n = 11)	X^2^ (p)
AI% MVLL (%)	5.9 ± 9.4	7.0 ± 10.3	10.7 ± 6.7	2.316 (0.314)
AI% CMJ (%)	10.1 ± 6.1	10.9 ± 8.6	12.7 ± 8.9	0.327 (0.849)
AI% S10_run_ (%)	-2.2 ± 1.9	-2.7 ± 1.6	-2.7 ± 2.3	3.169 (0.205)
AI S10_drib_ (%)	-6.0 ± 9.7†‡	-5.1 ± 2.6	-3.9 ± 2.7	11.959 (0.003)*
AI% S10_drib_-Deficit (%)	22.0 ± 32.3†	16.0 ± 8.4	13.6 ± 10.6	6.883 (0.032)*
AI% COD_run_ (%)	-3.7 ± 3.2	-3.2 ± 2.2	-3.3 ± 3.0	3.249 (0.197)
AI% COD_run_-Deficit (%)	10.3 ± 14.6	12.1 ± 14.8	5.2 ± 9.4	3.856 (0.145)
AI% COD_drib_ (%)	-10.4 ± 4.9†‡	-6.2 ± 3.0¢	-4.2 ± 3.1	28.695 (< 0.0001)*
AI% COD_drib_-Deficit (%)	33.9 ± 16.7†‡	18.1 ± 9.9	12.1 ± 11.6	26.958 (< 0.0001)*
AI% Balance (%)	1.9 ± 2.2	1.7 ± 2.5	7.5 ± 10.4¥§	6.946 (0.031)*

All values are expressed in median ± interquartile range. Negative AI% values represent better performance on the right side in sprint and COD tasks. PHV: peak height velocity; X^2^: Chi-square values obtained from the Kruskal-Wallis test; LLMV: lower limb muscle volume; AI%: asymmetry index percentage; CMJ: countermovement jump; S10_run_: linear sprint of 10 m without the ball; S10_drib_: linear sprint of 10 m with the ball; COD_run_: change of direction without the ball; COD_drib_: change of direction with the ball. * biological maturity status effect. † significantly different from Circa-PHV; ‡ significantly different from Post-PHV; ¢ significantly different from Post-PHV; ¥ significantly different from Pre-PHV; § significantly different from Circa-PHV.

In Table 4, the kappa coefficients indicated agreement between S10_drib_ and S10_drib_-Deficit was perfect in Pre-PHV players. Additionally, the highest agreement between asymmetry indices of S10_drib_ and COD_drib_ and between S10_drib_-Deficit and COD_drib_ was found in Pre-PHV players.

**TABLE 4:** Kappa coefficients and accompanying descriptors for levels of agreement describing how consistently asymmetry favored the same side in biological maturity groups (Pre-, Mid- and Post-PHV, and pool group).

Biological maturity groups	AI% S10_drib_-Deficit (%)	AI% S10_drib_-Deficit (%)	AI% COD_drib_ (%)	AI% COD_drib_-Deficit (%)
AI% S10_drib_ (%)	Pre-PHV	1.00 (perfect)	0.44 (moderate)	0.35 (fair)
	Circa-PHV	0.52 (moderate)	0.32 (fair)	0.32 (fair)
	Post-PHV	0.81 (almost perfect)	0.21 (fair)	-0.03 (slight)
	Pool group	0.80 (substantial)	0.26 (fair)	0.19 (slight)

AI% S10_drib_-Deficit (%)	Pre-PHV	–	0.44 (moderate)	0.35 (fair)
	Circa-PHV	–	-0.14 (slight)	-0.14 (slight)
	Post-PHV	–	0.07 (slight)	-0.14 (slight)
	Pool group	–	0.18 (slight)	0.10 (slight)

AI% COD_drib_ (%)	Pre-PHV	–	–	0.90 (almost perfect)
	Circa-PHV	–	–	0.73 (substantial)
	Post-PHV	–	–	0.80 (substantial)
	Pool group	–	–	0.82 (almost perfect)

PHV: peak height velocity; AI%: asymmetry index percentage; S10_drib_: linear sprint of 10 m with ball; COD_drib_: change of direction with ball.

## DISCUSSION

We aimed to determine whether biological maturation status affects asymmetry in LLMV, linear and COD sprints (with and without the ball), vertical jumping, and dynamic balance in young male soccer players. We also evaluated the agreement between asymmetries in linear dribbling, COD_drib_, linear dribbling deficits, and COD_drib_ deficits across different stages of maturation in these players. The Pre-PHV players showed the highest asymmetry in S10_drib_, COD_drib_, and COD_drib_-Deficit, which decreased with maturation. However, Post-PHV players showed increased balance asymmetry compared to their younger counterparts. A fair to moderate agreement was observed between S10_drib_ and COD_drib_ asymmetry indices, with younger players showing more consistency in dribbling tasks and their corresponding deficits. The agreement between S10_drib_-Deficit and COD_drib_ was moderate in Pre-PHV and slight in Circa- and Post-PHV.

Our findings confirmed that biological maturation significantly influences inter-limb asymmetry in soccer-specific tasks. Pre-PHV players exhibited the greatest asymmetry in S10_drib_, COD_drib_, and the respective deficits. As maturation progresses, biological growth and structured training reduce asymmetries in dynamic actions like dribbling [1]. These adaptations could be enhanced due to coordination and neuromuscular stability as seen in elite youth players [5]. To address this issue, our findings indicated that coaches are encouraged to guide the Pre-PHV young male soccer players balancing the dribbling skill acquisition between the dominant and non-dominant limbs. The bilateral practice can reduce the lateral asymmetry in soccerspecific tasks during the developmental stage [35].

Notably, we found that S10_run_ and COD_run_ asymmetry remained stable across maturity groups. This finding was in line with Loturco et al.’s study [9] reported that asymmetry during linear sprinting has less impact on performance than in sport-specific actions. This suggests that straight-line running is less sensitive to neuromuscular maturation.

Interestingly, Post-PHV players showed increased balance asymmetry compared to Pre- and Circa-PHV groups. This finding was possibly related to rapid growth, temporarily disrupting neuromuscular control and impairing postural coordination [14, 36, 37]. This maturational imbalance, marked by altered limb proportions and proprioceptive lag, may reduce motor control despite strength gains [38, 39]. Neuromuscular training focusing on proprioception, unilateral balance, and dynamic stability can help to reduce balance asymmetry and injury risk [10, 40]. This notion is essential to youth players during growth spurts when injury incidence is elevated [41]. Systematic screening and timely intervention are recommended to support long-term athletic development.

The absence of CMJ asymmetry across maturation groups aligns with the literature. Gonzalo-Skok and Bishop [1] reported consistent unilateral jump asymmetries (9–13%) in youth football players regardless of maturation stage. This stability may reflect to (1) compensatory neuromuscular mechanisms in bilateral tasks [10], (2) the moderating influence of sport-specific training during adolescence [39], (3) and/or the complex interaction between physical growth and motor skill development [38]. Moreover, CMJ measure for asymmetry may have low sensitivity to maturity-related neuromuscular differences in youth athletes [13]. Although the asymmetries observed in our study (10.1–12.7%) were not approached to a significant level, the practical implications for long-term athlete development, injury prevention [7], and individualised training programmes should be considered in further reports.

No effects of maturation status on LLMV asymmetry in young male soccer players may be due to symmetrical muscle growth during adolescence [39] and/or the greater influence of sport-specific training [10]. Inter-individual variability in response to maturation and training [38] and methodologies (e.g. maturation classification and muscle measurement techniques [42] could also contribute to this observation. The choice of measurement technique and the specific muscle groups analysed (e.g., quadriceps and hamstrings, which generally develop symmetrically) may have influenced the LLMV results [43]. While the anthropometric method used in this study was a field-based setting, imaging techniques like magnetic resonance imaging (MRI) or dual-energy X-ray absorptiometry (DXA) can provide biomedical information. Its reliance on tissue density and limb geometry assumptions may introduce estimation errors, which should be considered when interpreting our findings.

The modest consistency observed between S10_drib_ and COD_drib_ asymmetry indices supports the findings of Scinicarelli et al. [6] showing that asymmetries do not necessarily transfer across tasks but emphasising their task-specific nature. Limited agreement between dribbling and COD asymmetry deficits further highlights their distinct neuromuscular demands. Indeed, dribbling skills require multi-planar coordination, visual-motor integration, and fine motor control. Conversely, COD relies principally on eccentric strength and force application mechanics [3]. From a biomechanical standpoint, COD involves greater braking and propulsive forces, rapid deceleration-acceleration transitions, and increased reliance on lateral stability and limb stiffness. Likewise, soccer dribbling skills emphasise ankle-foot coordination and rhythmic limb control [44]. These biomechanical divergences likely reduce the overlap in neuromuscular strategies employed, thereby lowering asymmetry agreement between tasks [45]. Therefore, asymmetry trends should be assessed based on specific performance demands rather than generalised movement patterns. Interestingly, our findings indicate higher agreement in Pre-PHV players, likely reflecting their generalised asymmetry across multiple tasks due to underdeveloped motor coordination. This supports that younger athletes exhibit global motor control deficits rather than task-specific discrepancies [46]. Conversely, Post-PHV players showed lower agreement levels between asymmetry indices. These findings supported the notion that task-specific adaptations become more prominent with maturation, reducing inter-task agreement in asymmetry measures [13, 14]. This reflects an increasing specialisation of neuromuscular control, where athletes adopt distinct limb-dominant strategies tailored to the biomechanical requirements of each task [45]. However, we observed strong agreement between linear dribbling and linear dribbling deficit asymmetry indices, consistent with Pleša et al. [14] reported inter-task agreement when biomechanical and neuromuscular demands overlap. Additionally, the substantial agreement observed between COD_drib_ and its deficit asymmetry indices across all groups aligns with Loturco et al. [9]. These indices may better reflect inter-limb performance disparities by minimising the confounding effects of technique or external variables. Incorporating biomechanical principles and neuromuscular specificity is essential for accurately interpreting asymmetry patterns and tailoring individualised performance and injury prevention strategies [47].

Our study offers a novel finding to simultaneously assess multiple inter-limb asymmetry indicators (e.g., sprinting, COD, dribbling, balance, and LLMV) across defined biological maturity stages. This multidimensional framework provides a comprehensive and ecological understanding of evolving asymmetry patterns during youth development. Our findings reveal three key insights: (1) asymmetry magnitude and direction vary across tasks and maturity levels, with younger youth players exhibiting greater asymmetry in technical tasks like dribbling, while older youth players show more balance-related asymmetries; (2) younger players (Pre-PHV) display more consistent asymmetry direction across tasks, suggesting generalized asymmetries early in development, whereas older players demonstrate task-specific adaptations; and (3) the disconnect between muscle volume asymmetry and functional performance underscores the greater influence of neuromuscular control and motor skill proficiency over structural differences.

These findings highlight the importance of early identification and management of asymmetry through age- and maturation-specific training strategies. The pronounced asymmetries observed in Pre-PHV players suggest that this stage represents a critical window for screening imbalance patterns using multi-task assessments (e.g., combining dribbling and balance tests). Training during this period should prioritise neuromuscular control, movement quality, and coordination through unilateral stabilisation and bilateral motor skill development. As players progress to Circa- and Post-PHV stages, programs can progressively incorporate task-specific drills, unilateral strength exercises, and balance training to address sport-specific demands and refine functional symmetry. This phased approach supports long-term athlete development by optimising adaptation to biological growth for reducing injury risk.

Notably, Monasterio et al. [41] found that overall injury incidence and burden were significantly higher in pre-PHV players with accelerated growth rates, compared to peers with lower or moderate growth rates. This highlights the importance of early asymmetry identification and intervention as injury prevention strategies. Regular asymmetry assessments can guide individualised interventions, supporting more effective athlete development. Future research should explore how asymmetry evolves throughout maturation to better understand its persistence, adaptability, and the relationship between injury occurrence and long-term performance. These recommendations are further supported by Souissi et al. [40], who demonstrated that targeted training programs significantly enhance functional performance and muscle power after ACL reconstruction. This underscores the importance of reducing asymmetries in athletic development.

This study has some limitations. Firstly, its cross-sectional design prevents causal conclusions about the relationship between biological maturation and performance asymmetries. Longitudinal studies tracking the same athletes through maturation stages would offer deeper insights. Secondly, while field-based assessments were valid and reliable, integrating biomechanical analyses (e.g., motion capture or force plates) could reveal the neuromuscular mechanisms underlying the observed asymmetries. Thirdly, the study sample pool was limited to regional-level adolescent players, restricting generalizability to elite and sub-elite levels. Fourthly, future research should specifically address adolescent girls, as sex differences in growth patterns may influence asymmetry and skill acquisition. Expanding research to include female athletes and varying competitive levels would enhance the applicability of findings. Lastly, potential confounding variables such as playing position, training volume, and training experience were not controlled. These factors may influence asymmetry patterns due to differences in physical demands, technical requirements, and exposure levels. Future studies should consider these variables to improve the interpretability of asymmetry outcomes.

## CONCLUSIONS

We highlighted the influence of biological maturation on inter-limb asymmetry during sport-specific tasks in young soccer players, particularly dribbling and COD movements. Our study emphasises the need for maturity-sensitive assessments and suggests that practitioners should avoid generalising asymmetry profiles across tasks. Coaches should consider maturity status and use task-specific assessments for asymmetry screening to optimise individualised training programmes.

## Data Availability

Data are available from the first author upon reasonable request.
